# Updated Review and Meta-Analysis of Probiotics for the Treatment of Clinical Depression: Adjunctive vs. Stand-Alone Treatment

**DOI:** 10.3390/jcm10040647

**Published:** 2021-02-08

**Authors:** Viktoriya L. Nikolova, Anthony J. Cleare, Allan H. Young, James M. Stone

**Affiliations:** 1 Centre for Affective Disorders, Institute of Psychiatry, Psychology & Neuroscience, King’s College London, London SE5 8AF, UK; anthony.cleare@kcl.ac.uk (A.J.C.); allan.young@kcl.ac.uk (A.H.Y.); j.stone@bsms.ac.uk (J.M.S.); 2 National Institute for Health Research Biomedical Research Centre at South London and Maudsley NHS Foundation Trust and King’s College London, London SE5 8AF, UK; 3 South London and Maudsley NHS Foundation Trust, Bethlem Royal Hospital, Beckenham BR3 3BX, UK; 4 Brighton and Sussex Medical School, Brighton BN1 9PX, UK

**Keywords:** depression, probiotics, systematic review, meta-analysis

## Abstract

Recent years have seen a rapid increase in the use of gut microbiota-targeting interventions, such as probiotics, for the treatment of psychiatric disorders. The objective of this update review was to evaluate all randomised controlled clinical trial evidence on the efficacy of probiotics for clinical depression. Cochrane guidelines for updated reviews were followed. By searching PubMed and Web of Science databases, we identified 546 new records since our previous review. A total of seven studies met selection criteria, capturing 404 people with depression. A random effects meta-analysis using treatment type (stand-alone vs. adjunctive) as subgroup was performed. The results demonstrated that probiotics are effective in reducing depressive symptoms when administered in addition to antidepressants (SMD = 0.83, 95%CI 0.49–1.17), however, they do not seem to offer significant benefits when used as stand-alone treatment (SMD = −0.02, 95%CI −0.34–0.30). Potential mechanisms of action may be via increases in brain-derived neurotrophic factor (BDNF) and decreases in C-reactive protein (CRP), although limited evidence is available at present. This review offers stronger evidence to support the clinical use of probiotics in depressed populations and provides an insight into the mode of administration more likely to yield antidepressant effects.

## 1. Introduction

Major depressive disorder (MDD) is a common, complex, and heterogeneous illness that is characterized by persistent low mood and anhedonia, and a combination of sleep disturbances, changes in appetite, feelings of worthlessness or guilt, poor concentration, and suicidal ideation [1]. MDD significantly diminishes quality of life [2], and is currently the leading cause of disability worldwide [3]. It is estimated that more than 264 million people suffer from MDD globally, and there are approximately 800,000 suicide deaths yearly linked to depression [3].

It has been estimated that up to 60% of patients with MDD experience some degree of non-response to the wide range of pharmacological treatments [4], which predominantly target monoamine dysregulation in the brain. This suggests that there are other mechanisms implicated in the aetiology of depression, and abnormalities in the neuroendocrine, immune, neurotrophic, and metabolic systems have been identified [5]. Most recently, due to advances in metagenomic technologies, the gut microbiota has also been implicated in the pathophysiology of depression, via its complex bidirectional communication with the brain, also known as the gut–brain axis [6]. Gut dysbiosis (bacterial imbalance) and compositional differences have been identified in people with affective disorders, compared to healthy individuals, and these abnormalities have been linked to symptom severity [7,8,9,10], increases in pro-inflammatory cytokines and cortisol, and decreased in levels of brain-derived neurotrophic factor (BDNF) [11,12,13,14], among other factors, which are prominent biomarkers of depression. Consequently, there has been a surge in novel treatment paradigms targeting the gut microbiota in depressive disorders, including probiotics, prebiotics, nutraceuticals, and various dietary interventions.

Previously, we published a systematic review of randomised controlled trials (RCTs) testing the efficacy of probiotics for clinical depression [1]. We concluded that there was limited evidence to substantiate health claims in clinically depressed populations, due to the small number of high-quality studies at the time, although we tentatively suggested there may be a beneficial effect of probiotics on depressive symptoms when administered in addition to antidepressants. Similarly, an evaluation of potential mechanisms of action of probiotics was deemed premature. Due to the inconclusive findings of this review, and the numerous original articles published since in this rapidly developing field of research, an updated review is warranted. Further, other reviews that have been published have reported on the antidepressant properties of probiotics in a mixture of healthy populations, populations under stress, and populations with other primary psychiatric or non-psychiatric diagnoses (e.g., bowel disorders, fibromyalgia, neurodegenerative disorders, etc.) [15,16,17]. However, for probiotics to be considered a viable treatment option for depression, RCT evidence from clinically defined populations with depression rather than with other primary diagnoses is needed. The objective of this review was, therefore, to identify and evaluate all current evidence from RCTs on the efficacy of probiotics in reducing depressive symptoms among people with clinical depression.

## 2. Materials and Methods

We followed the Preferred Reporting Items for Systematic Reviews and Meta-Analyses (PRISMA) [18] guidelines, as well as guidance on performing updated reviews [19,20].

### 2.1. Search Details

We replicated the search strategy used in our earlier review [1]. The search was conducted in PubMed and Web of Science on 15 May 2020. The terms searched were: [probiotic* OR synbiotic* OR *Lactobacillus* OR *Bifidobacterium*] AND [depress* OR depression OR MDD or TRD] (all fields). The date of publication was limited from 11 April 2018 (exactly one month before the date of our previous search, to account for any potential missed records due to a delay in appearing on databases). The search was limited to human studies (database permitting) and English language. An additional search on Google Scholar was performed to identify any studies that may have been missed. This was further supplemented by reviewing the bibliographies of included studies and recent reviews.

### 2.2. Selection Criteria

Studies were considered eligible if (1) they were RCTs, in which probiotics were administered as the sole active intervention; (2) the effects of probiotics on depressive symptoms were assessed as the primary outcome using a validated measure; and (3) the presence of depression was ascertained either with a diagnostic interview or with the use of a validated scale. RCTs in which the population was selected for the presence of another primary condition (e.g., irritable bowel syndrome, obesity, migraine etc.) were not eligible for inclusion.

### 2.3. Data Extraction

For each RCT, we extracted study (author, year, country) and population (sample size, age, gender, method used to identify depression) characteristics. We also extracted information on trial design, intervention content, duration, tolerability and compliance, primary outcome measure, and any biomarkers assessed (e.g., inflammatory cytokines, BDNF, cortisol, etc.). Continuous data describing antidepressant treatment effect were obtained (i.e., pre- and post-treatment depression scores or change in depression score) and presented as standardised mean difference (Hedge’s *g*). Studies were also verified against their original entries on trial registries (where available) to evaluate completeness of reporting. Where post-hoc analyses were published, relevant data were extracted from these as well.

### 2.4. Quality Assessment

Included trials were assessed for risk of bias (RoB), using an adapted version of the Scottish Intercollegiate Guidelines Network (SIGN) tool, as previously described elsewhere [21]. Studies were evaluated on nine factors, including appropriateness of the research question, randomisation, concealment of allocation, blinding, comparability of groups at baseline, groups treated the same throughout (includes inter-site differences), use of standardised outcome measures, use of intent-to-treat analysis, and presence of for-profit bias (allegiance). Overall risk of bias was rated as low, moderate, or high.

### 2.5. Quantitative Synthesis

We performed a random-effects meta-analysis on Hedge’s *g* standardised mean difference (SMD) as the effect measure, applying the inverse-variance method. Analyses were performed using the meta package in R (version 4.0.0; R Foundation for Statistical Computing, Vienna, Austria). Standard deviations (SDs) for mean change were extracted where possible or extrapolated from standard error (95% confidence intervals or *p*-values). Inter-study heterogeneity was quantified using the DerSimonian–Laird estimator, and is reported with the *I*^2^ statistic, which represents the fraction of variation between studies attributable to heterogeneity. According to convention, a value around 25% indicates low heterogeneity, 50% is moderate, and 75% is high [22]. A significant *p*-value (<0.05) indicates the presence of heterogeneity. Effect size was categorized as small (SMD = 0.2), moderate (SMD = 0.5), or large (SMD = 0.8) [23].

Pre-planned subgroup analyses, derived from the insights of our earlier review, were performed to explore the effects of probiotics on depressive symptoms according to treatment method (i.e., add-on vs. stand-alone). Due to the low number of studies, and in accordance with published recommendations [24], we did not perform additional sensitivity analyses, meta-regression, or assessment of publication bias. However, publication bias was likely to have been low, as there were several studies reporting negative findings.

## 3. Results

### 3.1. Search Results

**Figure 1:**
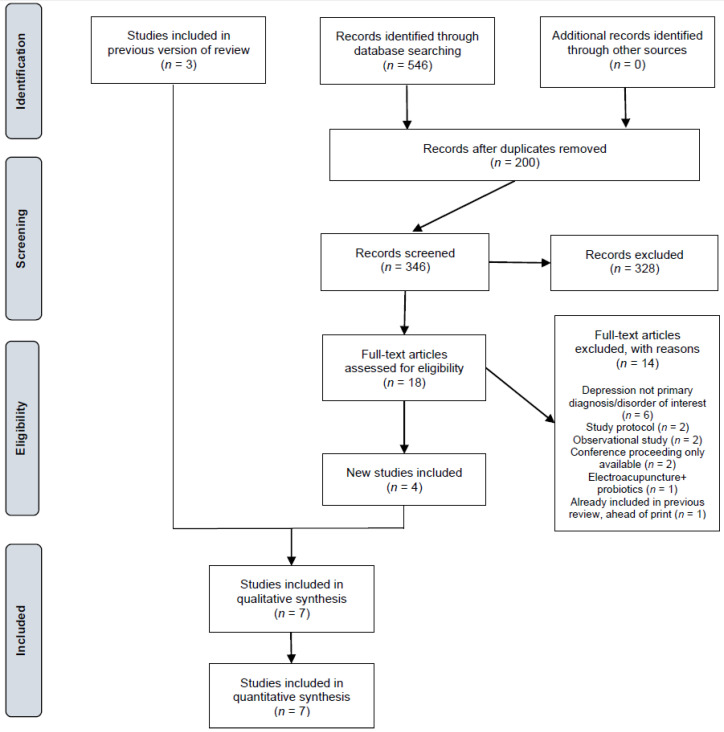
Preferred Reporting Items for Systematic Reviews and Meta-Analyses (PRISMA) study flow diagram for updated reviews. Adapted from Stovold et al. [25].

Our search identified 546 records, of which 200 were duplicates, leaving 346 uniquepublications. Of these, 328 were excluded as irrelevant upon review of the title/abstract. The full text of the remaining 18 was screened for eligibility, and 14 further articles were excluded (see Figure 1 for breakdown of reasons for exclusion). Four unique RCTs met selection criteria. These were combined with the three RCTs included in our previous review for the purposes of qualitative and quantitative synthesis.

### 3.2. Characteristics of Included Studies

Key characteristics of the seven studies are presented in Table 1. Overall, the seven RCTs captured 404 people with clinical depression from five countries (Iran, Poland, Japan, New Zealand, and Australia) who were randomised in a parallel group design to either probiotic/synbiotic or placebo/treatment as usual (TAU). The average age was similar across studies (35–43 years), with larger variation in gender distribution (52–85% female). The duration of the probiotic intervention was also similar: 8 weeks in six studies and 6 weeks in one study [26]. In terms of contents, all studies but one [27], used species from the Lactobacilli and Bifidobacteria genera; however, the species, strains, and dosage varied between studies. A key difference was the intervention mode chosen: two trials initiated probiotics in antidepressant-free populations, while the remaining five initiated probiotics in addition to antidepressants. Among these, participants in three studies were on stable medication and dosage for 4+ weeks prior to enrollment [27,28,29]; participants in one study had the antidepressant (fluoxetine) initiated as part of the study 4 weeks prior to starting on the probiotic [26]; and in one study, there was a mixture of both ongoing and newly initiated selective serotonin reuptake inhibitor (SSRI), although intervention groups did not differ on this parameter [30]. Studies also varied in terms of depression severity, ranging from mild/moderate to treatment-resistant. All studies used validated scales as the primary outcome measure, with four studies employing a self-rated measure (Beck Depression Inventory, BDI) and the remaining three employing a clinician-rated measure (Hamilton Depression Rating Scale (HAMD) or Montgomery–Asberg Depression Rating Scale (MADRS)). These three scales are the most widely used in the field, and their reliability and comparability are well described [31,32]. 

### 3.3. Quality Assessment

Overall, study quality was high, as six studies were rated as having low RoB and only one was rated as having high RoB [27]. This study raised multiple concerns, as it was an open-label study with an undescribed method of randomisation and probable commercial conflict of interest. We performed the meta-analysis with and without this study to evaluate its impact on our results. Issues raised in the remaining studies pertained to uncertainty regarding the similarity of groups as a baseline (two studies), and not using an intent-to-treat analysis approach (one study). The full quality assessment is available online as Table S1.

### 3.4. Efficacy of Probiotics for the Treatment of Depressive Symptoms

Overall, there was a positive effect of probiotics on depressive symptoms (SMD = 0.58, 95% CI = 0.19–0.97), with high and significant heterogeneity (*I*^2^ = 73%, *p* < 0.01).

**Figure 2:**
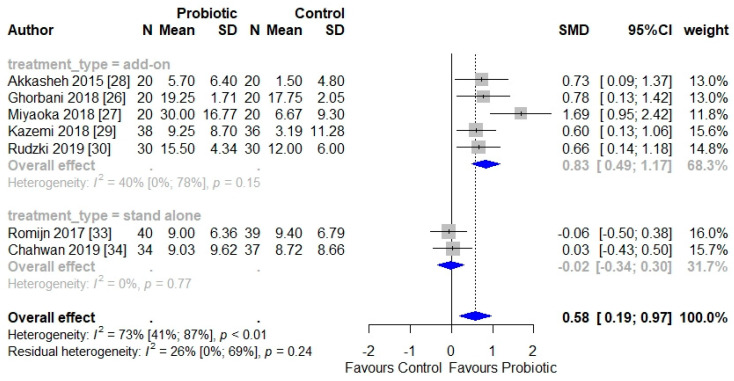
Forest plot of reduction in depressive symptoms post-treatment, grouped by treatment type (adjunctive vs. stand-alone).

When we investigated the efficacy of probiotics according to treatment type (add-on vs. stand-alone), the analysis demonstrated that probiotics significantly reduce depressive symptoms when administered in addition to antidepressants (SMD = 0.83, 95% CI = 0.49–1.17), but not when administered as stand-alone treatment (SMD = −0.02, 95% CI = 0.34–0.30) (Figure 2). Further, this sub-group analysis according to treatment type reduced heterogeneity considerably (*I*^2^ = 26%) and rendered it non-significant (*p* = 0.24).

We then removed the Miyaoka et al.’s [27] data from the analysis, to explore the individual impact of this study, which was highlighted as having a high RoB. The result remained significant, with a reduction in the size of the effect (SMD = 0.67, 95% CI = 0.40–0.95). Therefore, our findings indicate at least a moderate effect of probiotics in alleviating depressive symptoms when used as adjunctive treatment to an antidepressant.

### 3.5. Probiotics and Biomarkers of Depression

The effects of probiotics on known biomarkers of depression were assessed in five studies. The range of parameters evaluated was wide, and included inflammatory markers, BDNF, cortisol, kynurenine and tryptophan levels and associated ratios, metabolic markers, oxidative stress markers, vitamin D, leptin, and gut microbiota markers. Among those investigated in more than one study, pro-inflammatory cytokines interleukin (IL)-6 and tumor necrosis factor alpha (TNF-α) showed no significant differences between probiotic and placebo-treated participants post-intervention, while IL-1β was significantly reduced in one study [35]. However, this became non-significant after controlling for body mass index (BMI) and dietary intake. C-reactive protein (CRP) was significantly reduced in the probiotic arm (compared to placebo) in a study that applied probiotics as adjunctive therapy [28], but not in a study of medication-free participants [33]. Similarly, BDNF was found to be significantly increased following probiotic treatment only when this was applied as adjunctive [36], and not as stand-alone treatment [33]. Interestingly, an inverse correlation between increases in BDNF and depression severity were also reported in this study [36]. The tryptophane/kynurenine ratio was measured in two add-on studies, but only found to significantly differ in one, with a reduction in the probiotic arm [29]. Finally, two add-on studies assessed cortisol (urinary vs. plasma) and reported statistically non-significant differences. However, Kazemi et al. observed a reduction of 20% in the probiotic group (compared to no reduction in the placebo group), which they argued may be clinically significant [35]. The remaining results are summarized in Table 2. Meta-analyses were not performed, due to the small number of observations per parameter.

### 3.6. Tolerability and Compliance

Table 3 provides a breakdown of adherence, drop-out, and adverse event data from the seven trials. Overall, probiotics were well-tolerated, with no serious adverse events in any study. One study did not report adverse event (AE) data [28], while the remaining six RCTs stated that all AEs recorded were mild, transient, and did not lead to trial discontinuation. The most frequently reported side-effects were gastrointestinal, including bloating, nausea, constipation, diarrhoea, and cramps. Also reported in more than one study were changes in appetite, dry mouth, and headache. Attrition rates were similar between the treatment and control groups in all studies. However, two studies (Chahwan et al. and Kazemi et al.) reported a high drop-out rate of nearly 30%, which was attributed to burdensome visit frequency in one of the studies. Regarding adherence, 5/7 studies explicitly stated this was monitored, but only three reported actual data.

## 4. Discussion

In this review, we aimed to evaluate all up-to-date evidence from RCTs on the efficacy of probiotics as a treatment for depressive symptoms among people with clinical depression without another primary diagnosis. Our analysis of the seven identified trials, capturing just over 400 depressed individuals, demonstrated that probiotics significantly reduce depressive symptoms after eight weeks of use, but only when used in addition to an approved antidepressant. The current limited evidence does not support the use of probiotics as a stand-alone treatment for depression.

At present, it is challenging to explain why probiotics may be effective as adjunctive therapy only. Largely, this is due to our poor understanding of the impact of antidepressants themselves on the gut microbiota. Here, we discuss two potential explanations: synergistic effect and additive effect. Studies have now established that antidepressants from most classes (including SSRIs, SNRIs, tricyclic antidepressants (TCA), monoamine oxidase inhibitors (MAOI), atypical and NMDA receptor antagonists) exhibit antimicrobial activity; however, the nature of this activity may differ between classes [38]. Conversely, some antibiotics also present antidepressant properties, and their efficacy for treatment-resistant depression is currently under investigation (e.g., minocycline [39,40]). It is yet unclear how the compositional gut microbiota abnormalities identified in depression [7,8] are impacted by these treatments. However, the long-term use of antidepressants with antimicrobial properties (which is commonly the case) likely leads to the development of adaptive alterations in the microbiota [38]. It is yet unclear whether this is to be considered an adverse effect or a mechanism of antidepressant action in the gut [38]. For example, chronic use of certain antidepressants with antimicrobial properties has been linked to microbial resistance [41], as depressed adults taking these medications seem to be more likely to develop *C. difficile* infections [42], and might also explain some commonly reported gastrointestinal side-effects and comorbidities (e.g., irritable bowel syndrome (IBS). Additionally, Flowers et al. [43] found that in adults with bipolar disorder atypical antipsychotics significantly decreased bacterial species known to have beneficial anti-inflammatory properties. Maintenance of a neuroinflammatory state in psychiatric disorders has previously been linked to gut dysbiosis [13]. Therefore, probiotics may exert their therapeutic benefits by restoring microbial balance in the gut, and also by minimising gastrointestinal complaints in these patients, allowing for the effects of antidepressant medication to not be dampened. On the other hand, it has also been proposed that antidepressants may themselves exhibit some restorative effects to the gut microbiota, impacted by prolonged or severe depressive illness [41], and that their antimicrobial effects differentially affect genera that are commonly correlated with human health and dysbiosis [38], thus providing an improved opportunity for probiotic supplements to exert their beneficial effects. For example, a recent study in rats found that the NMDA receptor antagonist ketamine amplifies levels of the genus *Lactobacilli* [44], which has been found to be reduced in depressed animals, with some evidence of decreases in clinical populations as well [45,46]. Further studies are needed to understand the possible synergistic interaction of probiotics and antidepressants in depression.

Alternatively, it has been proposed that probiotics may be effective for subtypes or symptoms of depression where conventional antidepressants have limited efficacy. For example, SSRIs have been found to be less effective for the treatment of anhedonia [47,48] and somatic presentations [49], while both SSRIs and SNRIs have shown limited efficacy for cognitive impairment [47,50] and anxious depression [51]. Simultaneously, animal models have identified various probiotics from the *Lactobacilli* and *Bifidobacteria* genera to be effective against each of these symptoms/sub-types (for a recent review, see [52]). Indeed, one of the RCTs included in this review, which applied a multi-strain probiotic as stand-alone treatment, reported significantly reduced cognitive reactivity to a sad mood, while overall depression was not improved [34]. This suggests probiotics may have an additive effect, by positively impacting symptoms that typical antidepressants are less effective against. However, not all commonly used depression rating scales assess these symptoms (e.g., the Patient Health Questionnaire (PHQ-9), Hospital Anxiety and Depression Scale (HADS)), and even those that include some items evaluating these symptoms (e.g., MADRS, HAMD-17, BDI), have been criticised for lack of sensitivity towards atypical presentations [53], as they do not cover the full range of symptoms and measure only unilateral change (e.g., decreased sleep, decreased appetite). Therefore, the selection of outcome measures that cover the full range of depressive symptoms may be most appropriate in this field of research.

Nevertheless, our finding is inconsistent with a plethora of pre-clinical findings, where probiotics have exhibited antidepressant properties in the absence of another treatment [11]. This translational gap may be due to the larger complexity and inter-personal variation in the gut microbiota in humans, and its susceptibility to influences from a wide range of factors, many of which cannot be controlled within clinical trials. A further source of variance stems from the wide array of symptoms and presentations covered under the umbrella term “depression”.

Despite the limited overlap between current clinical trials, two potential candidate mechanisms of action of probiotics as adjunctive therapy may be emerging from present evidence: an increase in BDNF and decrease in CRP levels. Significant differences for these biomarkers were reported in the probiotic group post-intervention only in studies with medicated participants. Attenuations in serum levels of the growth factor BDNF have been linked to depression, and importantly, to antidepressant response [5]. Indeed, the study that found a significant increase of BDNF in the probiotic group compared to the placebo group also reported a significant correlation between increases in BDNF and BDI score reduction [36]. Both RCTs that measured BDNF used the same probiotic strains (*L. helveticus + B. longum*), which have previously been linked to BDNF increases in a mouse model of depression [14]. Other probiotic formulations have also been found to increase hippocampal BDNF in mice [12,54,55]. CRP is a marker of chronic inflammation that appears frequently and reliably elevated in depressed populations [5]. A study of antidepressant non-responders reported that CRP was elevated in nearly half the population [56]. This suggests that probiotics may be contributing to the antidepressant response by reducing levels of CRP. Multiple animal and healthy volunteer studies have demonstrated that probiotics and the gut microbiota can alter circulating levels of pro-inflammatory and anti-inflammatory cytokines [13]. However, no significant changes in other inflammatory markers were detected in any of the RCTs. Unfortunately, the included studies did not report depression subtype, and inflammatory profiles are suggested to differ between subtypes [57]. Thus, further studies are needed to evaluate the anti-inflammatory potential of probiotics in clinical depression. Other mechanisms of action explored were metabolic markers, cortisol, markers of oxidative stress, and tryptophan metabolism; however, the evidence for these was either inconclusive or limited (i.e., measured only in one study). An important objective for future research is to understand the impact of probiotics on key biomarkers of depression in clinical populations, as well as the relationship with treatment response.

In terms of optimal probiotic supplement content and dosage, there is little consensus at present, as reflected by the trials captured in this review. As extensively discussed elsewhere, different probiotics may be more beneficial for certain depressive symptoms or subtypes [52]; however, RCTs in depression usually do not report symptom-level data, thus making it difficult to establish strain-specific effects in clinical populations. While efforts to delineate the contributions and function of individual probiotics in single-strain, pre-clinical studies have been made, multistrain formulations have shown higher potency in humans, and are suggested to exhibit synergistic effects, with an expanded benefit on host physiology [52]. Therefore, a reasonable and simultaneously cost-effective strategy seems to be the use of a multi-strain probiotic. Nevertheless, the selection of intervention contents needs to be evidence-based. Finally, while probiotics seem to be very well tolerated in the short term, the long-term safety and efficacy of these interventions remains to be evaluated, along with an appropriate follow-up to assess relapse rates.

One limitation of this review was the small number of studies included. While other reviews have captured larger numbers of studies, these have also included healthy volunteers and populations with a primary condition other than depression, as well as no restrictions on trial design. We considered this approach to be of limited value in determining the clinical utility of probiotics for the treatment of depression, and therefore restricted our inclusion criteria to only capture RCT designs and populations meeting a clinical cut-off for depression with no other primary diagnosis. This limited the power of our analyses, but increased specificity. While our sub-group analysis on the basis of treatment mode (stand-alone vs. add-on) yielded a clear reduction in heterogeneity and rendered it non-significant, it remains possible that there were other sources of similarity between the two stand-alone studies that were unknown to the authors, and could provide an alternative explanation to our findings. This meta-analysis should be updated whenever sufficient new data become available. Another limitation stemming from the small number of studies was the inability to perform meta-analyses of potential mechanisms of action. Next, while we included only clinically depressed populations, the definition of depression we adopted was broad (i.e. beyond diagnosis based solely on a structured clinical review). This was deemed acceptable here, as clinically recognized cut-offs on validated depression scales were employed by studies and significant comorbidities were screened out. Nevertheless, higher clinical heterogeneity may have been present in studies employing this method of recruitment.

## 5. Conclusions

Our updated analysis demonstrates that probiotics are effective in reducing depressive symptoms when administered in addition to antidepressants; however, they do not seem to confer significant benefits when used as stand-alone treatment. The evidence summarized here supports the clinical use of probiotics in depressed populations and provides an insight into the mode of administration more likely to yield antidepressant effects. However, little remains known about the mechanisms underlying the antidepressant properties of probiotics and their interaction with antidepressants in clinically depressed populations. Further large efficacy trials simultaneously assessing the impact of probiotics on known biomarkers of depression are needed.

## Figures and Tables

**Table 1:** Summary of key characteristics of randomised controlled trials (RCTs) of probiotics for clinical depression (in chronological order).

Study (Year)	Country	Sample				Intervention				Primary
		Population, Definition	Severity	Sample Size	Avg. Age, % Female	Type	Duration	Probiotic (CFU/g)/ Dose	Control/ Blinding	Outcome Measure
Akkasheh (2015) [28]	Iran	MDD, DSM-IV	At least moderate	40	37 years (y) 85%	Add-on to SSRI (citalopram)	8 weeks	*L. acidophilus* (2 × 10^9^) *L. casei* (2 × 10^9^), *B. bifidum* (2 × 10^9^) 1 capsule daily	Placebo Double-blind	BDI
Romijn (2017) [33]	New Zealand	Depressive symptoms, QIDS-SR	At least moderate	79	35 y 79%	Standalone	8 weeks	*L. helveticus* + *B. longum* (≥2 × 10^9^) 1.5 g daily	Placebo Double-blind	MADRS
Kazemi (2019) [29]	Iran	MDD, ICD-10	Mild-moderate	74	36 y 71%	Add-on to SSRI or amitriptiline	8 weeks	*L. helveticus* + *B. longum* (≥2 × 10^9^) 5 g daily	Placebo Double-blind	BDI
Ghorbani (2018) [26]	Iran	MDD, DSM-5	Moderate	40	35 y 70%	Add-on to SSRI (fluoxetine)	6 weeks	MS probiotic ^1^, 500 mg + 100 mg prebiotic 1 capsule daily	Placebo Double-blind	HAMD-17
Miyaoka (2018) [27]	Japan	TRD, DSM-IV-TR	At least moderate	40	43 y 52%	Add-on to SSRI or SNRI	8 weeks	*C. butyricum* 60 mg daily	TAU Open label	BDI
Rudzki (2019) [30]	Poland	MDD, DSM-IV-TR	Moderate	60	39 y 71%	Add-on to SSRI	8 weeks	*L. plantarum* (10 × 10^9^) 2 capsules daily	Placebo Double-blind	HAMD-17
Chahwan (2019) [34]	Australia	Depressive symptoms, BDI	Mild–severe	71	36 y 69%	Standalone	8 weeks	MS probiotic ^2^ (2.5 × 10^9^), 4 g daily	Placebo Triple-blind	BDI

^1^ Contents of MS probiotic in Ghorbani et al. [26]: *L. casaei* = 3 × 10^8^, *L. acidofilus =* 2 × 10^8^, *L. bulgaricus* = 2 × 10^9^, *L. rhamnosus* = 3 × 10^8^, *B. breve =* 2 × 10^8^, *B. longum* = 1 × 10^9^, *S. thermophilus* = 3 × 10^8^. ^2^ Contents of MS probiotic in Chahwan et al. [34]: *B. bifidum*, *B. lactis* W51 & W52, *L. acidophilus*, *L.* brevis, *L. casei*, *L. salivarius*, and *Lactococcus lactis* W19 & W58 = 2.5 × 10^9^. MDD: major depressive disorder, TRD: treatment-resistant depression, HC: healthy control, ICD: International Classification of Diseases, DSM: Diagnostic and Statistical Manual of Mental Disorders, BDI: Beck Depression Inventory, MADRS: Montgomery–Asberg Depression Rating Scale, MS: multi-strain probiotic, HAMD-17: Hamilton Depression Rating Scale 17-item, QIDS-SR: Quick Inventory of Depressive Symptomatology Self-Report, M: mean, SD: Standard Deviation, CFU: colony-forming unit, TAU: treatment as usual; SSRI: selective serotonin reuptake inhibitor; SNRI: serotonin-norepinephrine reuptake inhibitor.

**Table 2:** Biomarkers assessed in the included studies and corresponding findings (most frequently measured first).

Parameter Assessed	Study	Outcome
Inflammatory markers (IL-1β, IL-6, TNF-α)	Romijn (2017) [33]	No significant differences.
	Kazemi (2019) [35]	No significant differences after adjustment for multiple covariates.
	Rudzki (2019) [30]	No significant differences.
CRP	Akkasheh (2015) [28]	Significantly reduced in the probiotic group compared to placebo post-treatment.
	Romijn (2017) [33]	No significant difference.
BDNF	Romijn (2017) [33]	No significant difference.
	Kazemi (2019) [36]	Significantly increased in the probiotic group compared to placebo post-treatment. This was significantly correlated with reduction in depressive scores.
Tryptophane/kynurenine ratio	Kazemi (2019) [29]	Tryptophan/kynurenine ratio significantly reduced in the probiotic group compared to placebo post-treatment.
	Rudzki (2019) [30]	No significant difference.
Cortisol (urinary) (plasma)	Kazemi (2019) [35]	Not statistically significant, but potentially clinically relevant reduction in the probiotic group compared to placebo post-treatment
	Rudzki (2019) [30]	No significant difference.
Metabolic markers (insulin, FPG, lipids, cholesterol)	Akkasheh (2015) [28]	Serum insulin and insulin resistance were significantly reduced in the probiotic group compared to the placebo group post-treatment.
Oxidative stress (tac, gsh)	Akkasheh (2015) [28]	GSH levels significantly increased in the probiotic group compared to the placebo group post-treatment.
Kynurenine	Rudzki (2019) [30]	Kynurenine significantly decreased in the probiotic group compared to the placebo group post-treatment.
Tryptophan	Rudzki (2019) [30]	No significant difference.
Other kynurenine ratios	Rudzki (2019) [30]	3HKYN/KYN ratio significantly decreased in the probiotic group compared to the placebo group post-treatment.
Other tryptophan ratios	Kazemi (2019) [29]	Tryptophan/isoleucine ratio significantly reduced in the probiotic group compared to the placebo group post-treatment.
Leptin	Kazemi (2019) [37]	No significant difference (*p* = 0.07).
Vitamin D	Romijn (2017) [33]	No significant difference.
Gut microbiota (diversity and abundance)	Chahwan (2019) [34]	No significant differences.

IL: interleukin, TNF-α: tumour necrosis factor alpha, CRP: C-reactive protein, BDNF: brain-derived neurotrophic factor, FPG: fasting plasma glucose, TAC: total antioxidant capacity, GSH: glutathione.

**Table 3:** Drop-out, tolerability, and compliance rates in the included studies.

Study	Drop-Out Rate (*n*/Total *n*)		Adherence % (Doses Taken)	Adverse Events *n* *	
	Probiotic	Control		Probiotic	Control
Akkasheh (2015) [28]	3/20	2/20	90%	nr	nr
Romijn (2017) [33]	7/40	3/39	97%	77	91
Kazemi (2019) [29]	10/38	10/36	92%	10	1
Ghorbani (2018) [26]	0/20	0/20	Not mentioned	13	3
Miyaoka (2018) [27]	0/20	0/20	Not mentioned	3	3
Rudzki (2019) [30]	10/40	9/39	Monitored, nr	5	7
Chahwan (2019) [34]	11/34	13/37	Monitored, nr	65	43

* No serious adverse events (AEs) were reported in any study. High numbers in some studies reflect use of different methodology for collecting AE data (e.g. checklist). nr = not reported.

## Data Availability

Not applicable.

## Supplementary Materials

The complete quality assessment is available as Supplementary Material 1 online at https://www.mdpi.com/2077-0383/10/4/647/s1. Table S1: Study quality assessment using the SIGN tool.
