# Supplementary material for: Updated Review and Meta-Analysis of Probiotics for the Treatment of Clinical Depression: Adjunctive vs. Stand-Alone Treatment
Source: J Clin Med. 2021 Feb 8;10(4):647. doi: 10.3390/jcm10040647 (PMC7915600; doi:10.3390/jcm10040647)
Supplement: Supplementary file 1 [file jcm-10-00647-s001.pdf]

Supplementary Material 1

Table S1. Study quality assessment using the SIGN tool.

Criterion	Akka-sheh 2015	Ro-mijn 2017	Kazemi 2019	Ghor-bani 2018	Miyaoka 2018	Rudzki 2019	Chahwan 2019
Appropriateness and focus of study question	+	+	+	+	+	+	+
Assignment to treatment groups is randomised	+	+	+	+	?	+	+
Adequate concealment of allocation	+	+	+	?	-	+	+
Patients, clinicians and assessors are blinded	+	+	+	+	-	+	+
Similarity of groups at baseline	?	-	+	+	?	+	+
Similarity of groups during treatment period	+	+	+	+	+	+	+
Use of standardised outcome measure	+	+	+	+	+	+	+
Intent-to-treat analysis	+	+	+	+	?	-	+
Allegiance effect minimised	+	+	+	+	-	+	?
**Overall assessment or risk of bias**	**low**	**low**	**low**	**low**	**high**	**low**	**low**

+ low risk, – high risk, ? unclear risk .
